# Exploring the roles of snoRNA-induced ribosome heterogeneity in equine osteoarthritis

**DOI:** 10.3389/fvets.2025.1562508

**Published:** 2025-07-10

**Authors:** Alzbeta Chabronova, Marie Walters, Sara Regårdh, Stine Jacobsen, Louise Bundgaard, James R. Anderson, Mandy J. Peffers

**Affiliations:** ^1^Department of Musculoskeletal and Ageing Science, Institute of Life Course and Medical Sciences, University of Liverpool, Liverpool, United Kingdom; ^2^Department of Veterinary Clinical Sciences, Faculty of Health and Medical Sciences, University of Copenhagen, Copenhagen, Denmark

**Keywords:** small nucleolar RNAs, snoRNAs, horse, synovial fluid, osteoarthritis, ribosome, rRNA modifications

## Abstract

**Introduction:**

Osteoarthritis (OA) is a degenerative joint disease that greatly contributes to equine morbidity and poor welfare. Changes in cellular protein expression programs fuel the development and progression of OA. Small nucleolar RNAs (snoRNAs) are emerging as important regulators of OA (patho)biology. SnoRNAs are short non-coding RNAs that guide post-transcriptional modifications (PTMs) of ribosomal RNA (rRNA) nucleotides, which impact ribosome function and thus cellular protein expression programs. There is only very limited data on snoRNAs in equine OA.

**Methods:**

In this study, we induced OA in horses (*n* = 9) using a well-established equine carpal osteochondral fragment model of OA. We collected synovial fluid (SF) before (Day 0) and after OA-inducing surgery (Day 28, Day 70). Using small RNA sequencing, we then measured snoRNA levels in SF.

**Results:**

We identified 229 snoRNAs across all samples of which 30 snoRNAs were significantly differentially expressed (DE) in Day 28 vs. Day 0 comparison, 22 snoRNAs in Day 70 vs. Day 0, and finally, 23 snoRNAs in Day 70 vs. Day 28. On Day 28, the majority of DE snoRNAs were upregulated when compared to Day 0. In contrast, the majority of DE snoRNAs on Day 70 were downregulated when compared to Day 0 and Day 28. Altogether, 44 snoRNAs were DE across different comparisons, the majority of which were canonical snoRNAs. We then mapped all the predicted PTMs guided by the DE snoRNAs within a 3D ribosome.

**Discussion:**

Several of these PTMs were located within functionally important ribosomal regions. This included helices H89–H91 of peptidyl transferase center, helices H37–H39 of A-site finger and B1a ribosomal bridge, helices H70–H71, 5.8S-28S junction, and lastly, helices h14 and H95 of GTPase-associated center. Altogether, our novel data show that snoRNAs are regulated in equine OA, highlighting their potential as early molecular biomarkers and therapeutic targets. Targeting snoRNA to modulate protein synthesis in OA joints could ultimately improve outcomes for OA-affected horses.

## 1 Introduction

Osteoarthritis (OA) is a degenerative joint disease that greatly contributes to equine morbidity and poor welfare (1–4). In 2013, almost 14% of horse owners reported OA-related issues in their horses and/or ponies in the UK (1). In the USA, up to 60% of equine lameness is related to OA (3). Another study showed that one-third of 2- and 3-year-old thoroughbred horses had metacarpophalangeal cartilage lesions and OA (5). OA is a complex disease that affects all tissues of the joint, including synovium, articular cartilage, and subchondral bone (6). The clinical manifestation includes joint effusion, joint thickening or bony changes, decreased range of motion, pain on manipulation, lameness, gait abnormalities or shortened stride, or reluctance to move. These symptoms are results of disrupted joint homeostasis, articular cartilage degeneration, synovial hyperplasia, osteophytes formation, and subchondral bone sclerosis (2, 6–8). OA is an active disease process, but molecular mechanisms driving its development and progression are still incompletely understood, and their comprehension is vital for the development of effective OA treatments.

Translation and its precise regulation are critical for cellular homeostasis (9). In OA, tissues within the joint undergo critical changes, which are fuelled by substantial adjustments in their protein expression profiles (10, 11). Dysregulation of the ribosome, a complex cellular nanomachine that translates genetic information from mRNAs and synthesizes proteins, has been implicated in OA (patho)biology (9). The eukaryotic ribosome consists of four ribosomal RNAs (rRNAs; 18S, 5.8S, 28S, and 5S), and ~80 core ribosomal proteins (RPs) organized into small ribosomal subunits (SSU) and large ribosomal subunits (LSU) (12). Several recent discoveries revealed that not all ribosomes are built the same, and there is a level of diversity in ribosome composition referred to as ribosome heterogeneity (see Glossary—Supplementary Table S1) (13–16). Post-transcriptional modifications [PTMs; 2′-*O*-methylation (2′-*O*-me) and pseudouridylation (ψ)] of rRNAs are a major source of ribosome heterogeneity (17–19). The site specificity of rRNA PTMs is guided by small nucleolar RNAs (snoRNAs). SnoRNAs are short non-coding RNAs (20, 21), which can be classified into two subfamilies: box C/D (SNORD) or box H/ACA (SNORA) based on the presence of specific conserved sequence motifs (“boxes”) and secondary structures. Together with specific proteins, snoRNAs form ribonucleoprotein complexes referred to as snoRNPs. SNORDs associate with methyltransferase Fibrillarin (FBL), SNU13, NOP58, NOP56 and SNORAs associate with pseudouridine synthase Dyskerin (DKC1), NHP2, NOP10, GAR1. Within a snoRNP, the specific snoRNA guides modification of its target rRNA nucleotide by base-pairing with the rRNA target sequence, while C/D box-specific enzyme FBL catalyzes its 2′-*O*-me or H/ACA box-specific DKC1 catalyzes its ψ (20, 21). SnoRNAs are emerging regulators of OA (patho)biology via their function in regulating ribosome heterogeneity and function (22–26). Two recent studies demonstrated that OA synovial fluid (SF) instigated site-specific changes in the rRNA 2′-*O*-me and ψ profiles in human primary chondrocytes *in vitro* (25, 26). Importantly, depletion of snoRNAs guiding these OA-regulated rRNA PTMs (SNORD71 guiding 5.8-Um14 and SNORA33 guiding 28S-ψ4966) altered ribosome function and translation, altogether promoting OA-relevant changes in the chondrocytes' proteome (25, 26). Furthermore, in addition to their canonical function in mediating rRNA PTMs, some snoRNAs have also non-canonical functions and regulate pre-rRNA processing and ribosome biogenesis (*e.g.*, U3), alternative mRNA splicing, processing, and editing, or modifications of other RNA species such as small nuclear RNAs, tRNAs, and mRNAs (27).

Differential expression (DE) of snoRNAs was reported in human aging and OA cartilage (22, 23), equine aging cartilage (28), equine OA SF (29), mouse OA joints and serum (24), and serum of patients with cruciate ligament injury (30). However, there is only very limited data on snoRNAs in equine OA, none of which has focused on the ribosome heterogeneity aspect of snoRNA function. In this explorative study, we measured snoRNA expression levels in SF isolated from horses before and after OA-inducing surgery (the clinical study was performed at Copenhagen University and approved by The Danish Animal Experimentation Inspectorate, #2017-15-0201-01314). We utilized a well-established equine carpal osteochondral fragment model of OA, in which OA was induced in the middle carpal joint by creating an 8 mm fragment on the dorsodistal aspect of the radial carpal bone during an arthroscopic procedure (2). We then identified snoRNAs DE in OA and mapped the rRNA PTMs guided by these DE snoRNAs within a 3D ribosome structure to hypothesize the roles of ribosome heterogeneity in equine OA.

## 2 Material and methods

### 2.1 Study design, sample collection, and pre-processing

The clinical study was performed at Copenhagen University and approved by The Danish Animal Experimentation Inspectorate, #2017-15-0201-01314. Nine skeletally mature Standardbred trotters (seven mares and two geldings, 2.5–7 years, 397–528 kg) were included in this study. At the inclusion, horses were clinically healthy based on clinical examination, a subjective examination including a flexion test, radiographic imaging, hematological and biochemical analysis of blood, and arthrocentesis of both middle carpal joints, including routine laboratory SF analysis (white blood cell count and total protein). OA was surgically induced in the left middle carpal joint using the osteochondral fragment model of OA (OAC), and the right middle carpal joint underwent sham surgery as described previously (2). Two weeks post-surgery, horses commenced exercise on a treadmill for 5 days a week. SF from both middle carpal joints was collected into EDTA (ethylenediamine tetraacetic acid) tubes on Day 0, Day 28, and Day 70 post-surgery. On day 49, one horse (horse 5) reached grade 4 lameness and was therefore euthanised following the predefined humane endpoint criteria (lame at walk, grade 4). SF (*n* = 26, missing Day 70 for horse 5) was centrifuged for 20 min at 1,000 × g and 4°C to remove cells and debris. The supernatant was collected and stored at −80°C before small RNA sequencing analysis. Radiological imaging and histology (haematoxylin and eosin and safranin O staining) were performed, and the results described (31).

### 2.2 Sample processing and RNA isolation

The SF (500 μL) was treated with a 1 μg/μL hyaluronidase (Hyaluronidase type IV, Sigma-Aldrich, Gillingham, UK) and filtered through a Costar^®^ Spin-X^®^ polypropylene microcentrifuge tube filter with 0.22 μm pore cellulose acetate membrane (Corning, Flintshire, UK). Total RNA was extracted and DNase-treated using miRNeasy serum/plasma Advanced Kit (catalog #217204, Qiagen, Manchester, UK) according to the manufacturer's instructions. RNA yields and quality were determined by a NanoDrop (ThermoFisher, UK) and validated using Qubit™ Flex Fluorometer (ThermoFisher, UK) and RIN scoring.

### 2.3 Small RNA sequencing

The RNA (100 ng/sample) was submitted to the Center for Genomic Research (University of Liverpool, Liverpool, United Kingdom) for small RNA sequencing. One sample (horse 9, Day 0) failed during laboratory processing, and 25 samples thus completed small RNA sequencing. TAP decapping was used to remove 5′CAPs on some snoRNAs to reduce bias. The library was prepared with the NEBNext^®^ Small RNA Library Prep Set (New England Biolabs, Ipswich, MA, USA) with the addition of a Cap-Clip Acid Pyrophosphatase (Cell script, Madison, WI, USA) step to remove potential 50 caps found on some snoRNAs. Sample quantity and quality were determined using Qubit assay (catalog #Q33230, ThermoFisher, UK) and DNA high-sensitivity bioanalyser chip (catalog #5067-4626, Agilent, UK). Samples were pooled (equimolar ratios, 3% cassette marker F selecting range 120–160 bp) and small RNAs were sequenced using Illumina HiSeq4000 using 2 × 150 bp ends. Sequencing depth across samples varied, with total reads ranging from ~10 million to over 206 million per sample. Trimmed mean of M-values method (TMM) was used to address the variation in sequencing reads between samples. Data scaling and index de-multiplexing were undertaken using CASAVA version 1.8.2 (Illumina). Quality control was performed at several stages. Pre-sequencing: RNA quantity and quality were assessed using Qubit, Bioanalyzer. Post-sequencing: raw reads were processed using a quality control pipeline; adapters trimmed using Cutadapt v1.2.1; low-quality bases removed using Sickle v1.200 (minimum quality score of 20); reads <15 bp were discarded. PCA and correlation analyses were used to identify outliers. Read length distributions and mapping statistics were also analyzed to ensure quality and consistency. The equine genome was used as a reference and the sequences were mapped against the EquCab3.0. Alignment of reads was performed using TopHat version 2.1.0. Data normalization was performed as previously (28), using a generalized linear model (GLM) as it accurately models count data, efficiently handles complex experimental designs, and provides statistically robust and interpretable results. Random variations in the samples were formulated following negative binomial distributions (edgeR) and the false discovery rate was controlled using the Benjamini-Hochberg method. Variability was managed through standard filtering of low-abundance features and correction for differences in library size to avoid bias was calculated using the TMM method which are recommended best practices for small RNAseq datasets with limited replicates.

Differential expression (DE) analysis was performed in RStudio using edgeR (32). One sample (horse 5, Day 28) was identified as an outliner (deviated significantly in QC assessments) and therefore excluded from further analysis. GLM values were used to calculate log fold changes (logFC) for required groups using a likelihood ratio test. Small RNA sequencing data were deposited in ArrayExpress (accession E-TAB-11840).

### 2.4 Data analysis

Data analysis and graph-plotting were performed in RStudio (ggplot2, dplyr). Counts per million (CPM) reads are used in the figures in the results section. SnoRNAs with a *p*-value (p) < 0.05 and FC > 2 or FC < 0.5 were defined as significantly DE.

### 2.5 PTM mapping within the 3D ribosome structure

PTMs predicted to be guided by DE snoRNAs were mapped within a 3D structure of the human 80S ribosome [4UG0, (12)] using RiboXYZ (33).

## 3 Results

### 3.1 snoRNA expression profiles in osteoarthritic synovial fluid (OA SF)

**Figure 1:**
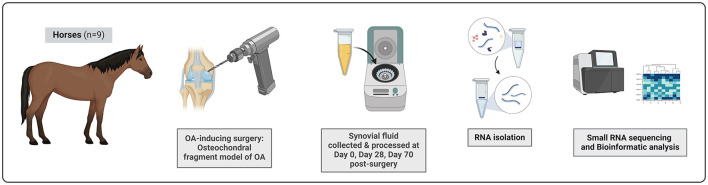
The experimental set-up. Nine skeletally mature clinically healthy Standardbred trotters were included in this study. OA was surgically induced in carpal joint using the osteochondral fragment model of OA. SF from both middle carpal joints was collected on Day 0, Day 28, and Day 70 post-surgery. Samples taken at Day 28 represent early stage of OA, Day 70 samples an advanced OA. SF was processed by centrifugation for 20 min at 1,000 × g and 4°C to remove cells and debris. SF samples were treated with hyaluronidase and total RNA was isolated. Small RNA sequencing was performed using the Illumina HiSeq4000 to measure snoRNA expression profiles in Day 0, Day 28, and Day 70 samples. The figure was created with BioRender.

To investigate changes in snoRNA expression profiles in SF during development of OA, we performed a small RNA sequencing analysis of the SF samples collected from horses (*n* = 9) before (Day 0) and longitudinally following the OA-inducing surgery (Day 28 representing earlier stages of OA, and Day 70 representing more advanced OA; Figure 1).

As expected, all horses developed OA by Day 70 as confirmed by radiological and histological changes in their joints (31). In OA joints, radiological imaging demonstrated osteophyte formation and bone proliferation and the OA-related radiographic changes ranged from mild to severe among individual horses. In line with this, histological scores of synovial tissue and articular cartilage samples collected at Day 70 were significantly higher in the OA joints compared to control joints. In OA samples, synovial tissue showed significantly greater cellular infiltration, intimal hyperplasia, and subintimal oedema, and articular cartilage presented with significantly greater chondrocyte necrosis, cluster formation, and focal cell loss scores.

**Figure 2:**
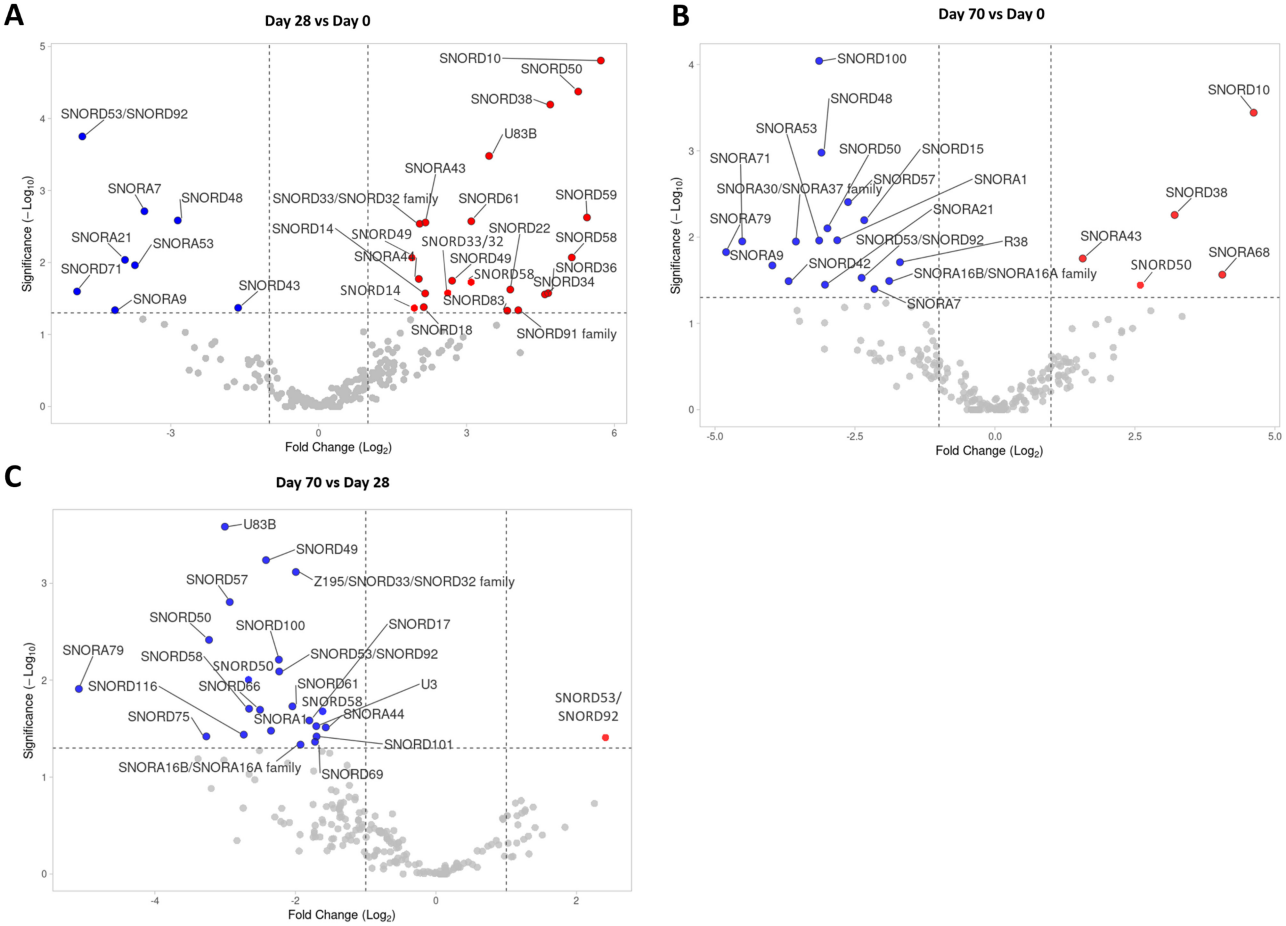
SnoRNA sequencing analysis of Day 0, Day 28, and Day 70 SF samples. **(A)** A volcano plot of snoRNAs identified in the Day 28 vs. Day 0 comparison, **(B)** in the Day 70 vs. Day 0 comparison, and **(C)** in the Day 70 vs. Day 28 comparison. Statistical significance was assessed by one-way ANOVA. The dotted lines represent cut-off values (FC > 2 and < 0.5; *p* < 0.05). Significantly downregulated snoRNAs are depicted in blue, significantly upregulated snoRNAs are in red.

SnoRNA expression in the SF samples collected on Day 0, Day 28, and Day 70 was assessed using small RNA sequencing analyses. In total, 229 snoRNAs were identified across all samples (Supplementary Table S2). Of these 229 snoRNAs, 30 snoRNAs were DE in the Day 28 vs. Day 0 comparison (Figure 2A); 22 snoRNAs were DE in the Day 70 vs. Day 0 comparison (Figure 2B); and finally, 23 snoRNAs were DE in the Day 70 vs. Day 28 comparison (Figure 2C). Table 1 lists all the DE snoRNAs for all comparisons, including the direction of the regulation, FC, and *p*-value. On Day 28, the majority of DE snoRNAs were upregulated when compared to Day 0. In contrast, the majority of DE snoRNAs on Day 70 were downregulated when compared to Day 0 and Day 28. Several snoRNAs were regulated at multiple time points. One snoRNA (SNORD53/92) was downregulated at all time points, five snoRNAs (SNORA7, SNORA9, SNORA21, SNORA45, and SNORD48) were decreased on Day 28 as well as Day 70 when compared to Day 0, and six snoRNAs (SNORA1, SNORA16A/B, SNORA79, SNORD50, SNORD57, and SNORD100) were all downregulated on Day 70 when compared to Day 0 as well as Day 28. For the upregulated snoRNAs, four snoRNAs (SNORA43, SNORD10, SNORD38, and SNORD50) were upregulated on Day 70 when compared to Day 0 as well as Day 28.

**Table 1:** List of identified DE snoRNAs for all comparisons.

**Name**	**Comparison**	**FC**	***p*-value**	**Direction**	**Family**	**Function**	**Target rRNA**	**Target rRNA nucleotide**	**Helix**	**Note**
SNORA1	Day 70 vs. Day 0	0.14	0.011	↓	H/ACA	Canonical	28S	U4471	H91	PTC
	Day 70 vs. Day 28	0.20	0.033	↓						
SNORA7	Day 28 vs. Day 0	0.09	0.002	↓	H/ACA	Canonical	28S	U1582, U1792	H35	.
	Day 70 vs. Day 0	0.22	0.040	↓					H38a	ASF
SNORA9	Day 28 vs. Day 0	0.06	0.046	↓	H/ACA	Canonical	28S	U1683, U1782	H37	.
	Day 70 vs. Day 0	0.06	0.021	↓					H38a	ASF
SNORA16B/A family	Day 70 vs. Day28	0.26	0.046	↓	H/ACA	Canonical	28S	U4442	H89/90	PTC
	Day 70 vs. Day0	0.27	0.032	↓						
SNORA21	Day 28 vs. Day 0	0.07	0.009	↓	H/ACA	Canonical	28S	U4431, U4500	H89	PTC
	Day 70 vs. Day 0	0.12	0.036	↓					H91	PTC
SNORA30/37 family	Day 70 vs. Day 0	0.09	0.011	↓	H/ACA	Canonical	28S	U4673	H97	.
SNORA38	Day 70 vs. Day 0	0.31	0.020	↓	H/ACA	Non-canonical	.	.	.	.
SNORA43	Day 70 vs. Day 0	2.96	0.018	↑	H/ACA	Non-canonical	.	.	.	.
	Day 28 vs. Day 0	4.49	0.003	↑						
SNORA44	Day 70 vs. Day 28	0.34	0.031	↓	H/ACA	Canonical	18S	U822, U686	h20	.
	Day 28 vs. Day 0	4.09	0.017	↑						
SNORA53	Day 28 vs. Day 0	0.08	0.011	↓	H/ACA	Non-canonical	.	.	.	.
	Day 70 vs. Day 0	0.11	0.011	↓						
SNORA68	Day 70 vs. Day 0	16.68	0.027	↑	H/ACA	Canonical	28S	U4423	H89	PTC
SNORA71	Day 70 vs. Day 0	0.04	0.011	↓	H/ACA	Canonical	18S	U406	h12	.
SNORA79	Day70 vs. Day 28	0.03	0.012	↓	H/ACA	Non-canonical	.	.	.	.
	Day 70 vs. Day 0	0.04	0.015	↓						
SNORD10	Day 70 vs. Day 0	24.59	0.000	↑	C/D	Canonical & Non-canonical	28S	C3808	H71	Intersubunit bridge B3
	Day 28 vs. Day 0	52.87	0.000	↑						
SNORD14	Day 28 vs. Day 0	3.84	0.043	↑	C/D	Canonical	18S	C462	h14	.
	Day 28 vs. Day 0	4.47	0.027	↑						
SNORD15	Day 70 vs. Day 0	0.20	0.006	↓	C/D	Canonical	28S	A3785	H70	.
SNORD17	Day 70 vs. Day 28	0.29	0.026	↓	C/D	Canonical	28S	U3818	H71	Intersubunit bridge B3
SNORD18	Day 28 vs. Day 0	4.38	0.042	↑	C/D	Canonical	28S	A1326	H25a	.
SNORD22	Day 28 vs. Day 0	14.80	0.024	↑	C/D	Non-canonical	.	.	.	.
SNORD33/32 family	Day 70 vs. Day 28	0.25	0.001	↓	C/D	Canonical	18S, 28S	18S-U1326 (RD33), 18S-G1328 (RD32), 28S-A1524 (RD32)	h34	.
	Day 28 vs. Day 0	4.14	0.003	↑						
	Day 28 vs. Day 0	6.14	0.027	↑					H32	
SNORD34	Day 28 vs. Day 0	24.10	0.028	↑	C/D	Canonical	28S	U2837	H48/61	.
SNORD36	Day 28 vs. Day 0	25.22	0.027	↑	C/D	Canonical	18S	A668	h19	.
SNORD38	Day 70 vs. Day 0	9.25	0.006	↑	C/D	Canonical	28S	A1871	H39	.
	Day 28 vs. Day 0	25.95	0.000	↑						
SNORD42	Day 70 vs. Day 0	0.08	0.032	↓	C/D	Canonical	18S	U116	h7	.
SNORD43	Day 28 vs. Day 0	0.32	0.043	↓	C/D	Canonical	18S	C1703	.	.
SNORD48	Day 70 vs. Day 0	0.12	0.001	↓	C/D	Canonical	28S	C1881	H39/40	.
	Day 28 vs. Day 0	0.14	0.003	↓						
SNORD49	Day 70 vs. Day 28	0.19	0.001	↓	C/D	Canonical	28S	C4456	H90	PTC
	Day 28 vs. Day 0	3.72	0.009	↑						
	Day 28 vs. Day 0	6.53	0.018	↑						
SNORD50	Day70 vs. Day 28	0.11	0.004	↓	C/D	Canonical	28S	C2861, G2876	H62, H62/63	.
	Day 70 vs. Day 0	0.13	0.008	↓^*^						
	Day 70 vs. Day 28	0.16	0.010	↓						
	Day 70 vs. Day 0	6.05	0.036	↑^**^						
	Day 28 vs. Day 0	38.47	0.000	↑						
SNORD53/92	Day 28 vs. Day 0	0.04	0.000	↓	C/D	Canonical	28S	C3869	H72	.
	Day 70 vs. Day 0	0.19	0.030	↓						
	Day 70 vs. Day 28	0.21	0.008	↓+						
	Day 70 vs. Day 28	5.33	0.039	↑++						
SNORD57	Day 70 vs. Day 28	0.13	0.002	↓	C/D	Canonical	18S	A99	h7	.
	Day 70 vs. Day 0	0.16	0.004	↓						
SNORD58	Day 70 vs. Day 28	0.16	0.020	↓	C/D	Canonical	28S	G4228	H38	.
	Day 70 vs. Day 28	0.33	0.021	↓						
	Day 28 vs. Day 0	8.53	0.019	↑						
	Day 28 vs. Day 0	35.14	0.009	↑						
SNORD59	Day 28 vs. Day 0	43.51	0.002	↑	C/D	Canonical	18S	A1031	h24	.
SNORD61	Day 70 vs. Day 28	0.24	0.019	↓	C/D	Canonical	18S	U1442	H39/40	.
	Day 28 vs. Day 0	8.55	0.003	↑						
SNORD66	Day 70 vs. Day 28	0.18	0.020	↓	C/D	Canonical	18S	C1272	h33	.
SNORD69	Day 70 vs. Day 28	0.30	0.043	↓	C/D	Canonical	28S	G4494	H91	PTC
SNORD71	Day 28 vs. Day 0	0.03	0.025	↓	C/D	Canonical	5.8S	U14	.	5.8-28S juction
SNORD75	Day 70 vs. Day 28	0.10	0.038	↓	C/D	Canonical	28S	C4054, G4499	H77, H92	PTC
SNORD83	Day 28 vs. Day 0	14.19	0.047	↑	C/D	Non-canonical	.	.	.	.
SNORD91 family	Day 28 vs. Day 0	16.56	0.046	↑	C/D	Canonical	28S	G4618	H95 (SRL)	GAC
SNORD100	Day 70 vs. Day 0	0.11	0.000	↓	C/D	Canonical	18S	G436	h13	.
	Day70 vs. Day 28	0.21	0.006	↓						
SNORD101	Day 70 vs. Day 28	0.31	0.038	↓	C/D	Non-canonical	.	.	.	.
SNORD116	Day 70 vs. Day 28	0.15	0.037	↓	C/D	Non-canonical	.	.	.	.
U3	Day 70 vs. Day 28	0.31	0.030	↓	C/D	Non-canonical	.	.	.	.
U83B	Day 70 vs. Day 28	0.12	0.000	↓	C/D	Non-canonical	.	.	.	.
	Day 28 vs. Day 0	10.98	0.000	↑						

* GeneID: 111,775,528;

** GeneID: 111,775,529; + GeneID: 111,768,255; ++ GeneID: 111,768,254. ASF, A-site finger; Gac, GTPase-associated center; PTC, peptidyl-transferase center; SRL, sarcin–ricin loop.

Some snoRNAs were identified multiple times, as these are transcribed from different gene loci (*e.g.*, SNORD50, or SNORD53/92). For the DE snoRNAs, these snoRNA “variants” were usually regulated in the same direction even though the magnitude of the regulation was slightly different *e.g.*, SNORD49 on Day 28 vs. Day 0 (Figure 2A), or SNORD50 on Day 70 vs. Day 28 (Figure 2C). In two cases, however, the snoRNA “variants” were regulated in the opposite direction, SNORD50 on Day 70 vs. Day 0 (Figure 2B) and SNORD53/92 on Day 70 vs. Day 28 (Figure 2C). In the case of opposite regulation, we listed geneIDs of these snoRNAs and the associated direction of the regulation in Table 1.

Altogether, we identified 44 snoRNAs to be DE in equine OA (across different comparisons), 13 H/ACA box snoRNAs, and 31 box C/D snoRNAs. The majority of the identified DE snoRNAs (34/44) were canonical snoRNAs which guide PTMs on rRNAs. These 34 canonical snoRNAs are predicted to guide PTMs of 42 rRNA nucleotides: 13 × ψ and 29 × 2′-*O*-me. Of these, 27 are located on 28S, 14 on 18S, and one on 5.8S rRNA. The predicted rRNA targets of the DE snoRNAs are listed in Table 1. The information regarding the FC difference, *p*-value, direction of the regulation, functions, predicted rRNA targets of canonical snoRNAs, and the locations of these PTMs within the 2D helix structure of ribosome is included.

### 3.2 Mapping of the PTMs guided by DE snoRNAs within the 3D ribosome structure

**Figure 3:**
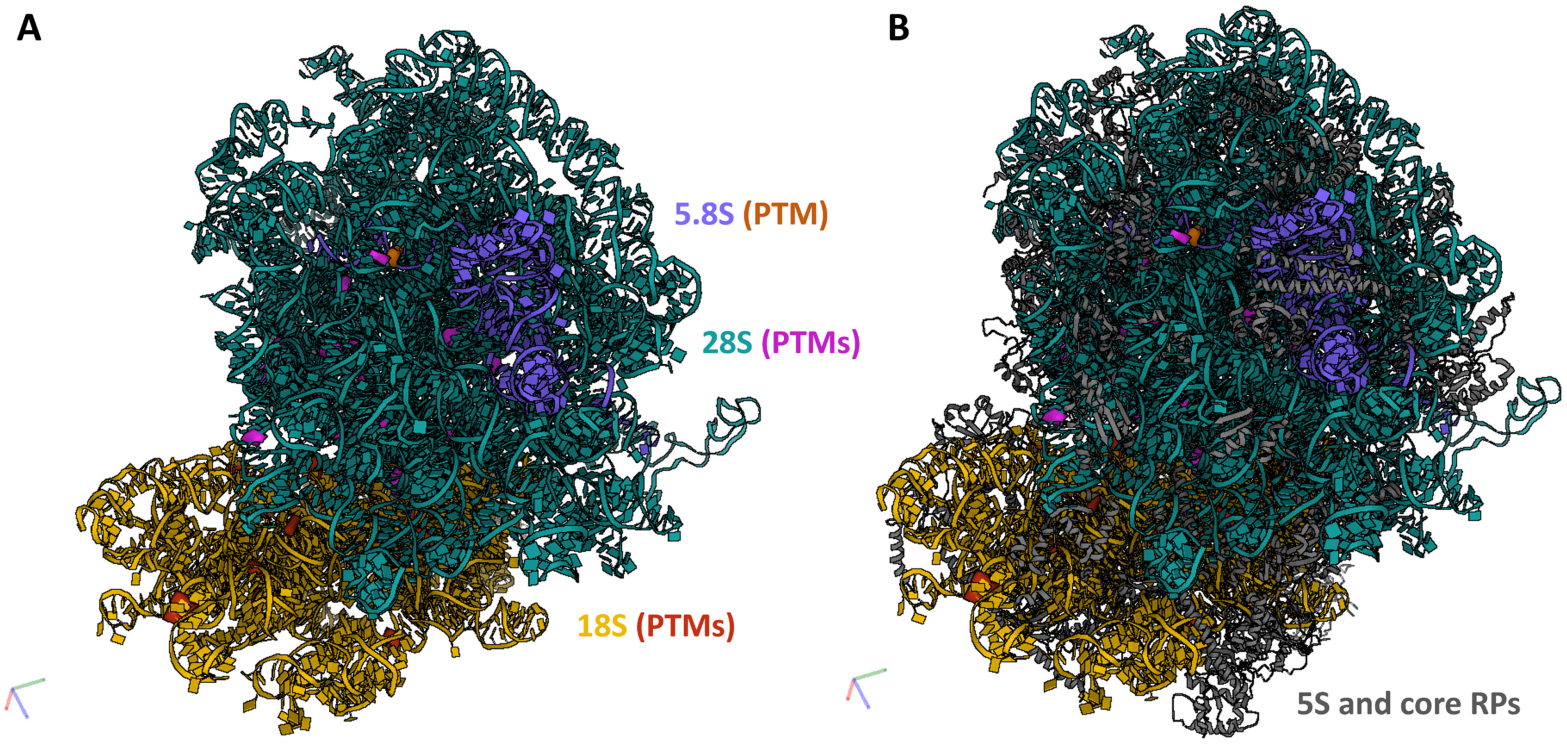
PTMs guided by the DE snoRNAs mapped within a 3D ribosome structure. To evaluate the potential of the identified OA-regulated canonical snoRNAs to affect ribosome function, we mapped their predicted PTMs within the 3D structure of the human ribosome (3D structure of the equine ribosome is not available). **(A)** In total, 42 PTMs were mapped on rRNAs, 18 on 18S rRNA, 27 on 28S rRNA, and one on 5.8S rRNA. **(B)** A localization of these PTMs within a full ribosome structure including 5S rRNA and core RPs. 18S rRNA (yellow), its modifications (red); 28S rRNA (teal), its modifications (purple); 5.8S rRNA (blue), its modification (orange); 5S rRNA and core RPs (gray). Human ribosome 3D structure (4UG0) was used for the visualization in the RiboXYZ database.

To hypothesize the potential of the OA-regulated canonical snoRNAs to affect ribosome function, we mapped the predicted 42 PTMs within the 3D structure of the eukaryotic ribosome (Figure 3). As a 3D structure of the equine ribosome is not available and there is high structural conservation across mammals, we used a human ribosome as a template (12). We employed the recently published RiboXYZ online database (33) for the PTM mapping and visualization.

**Figure 4:**
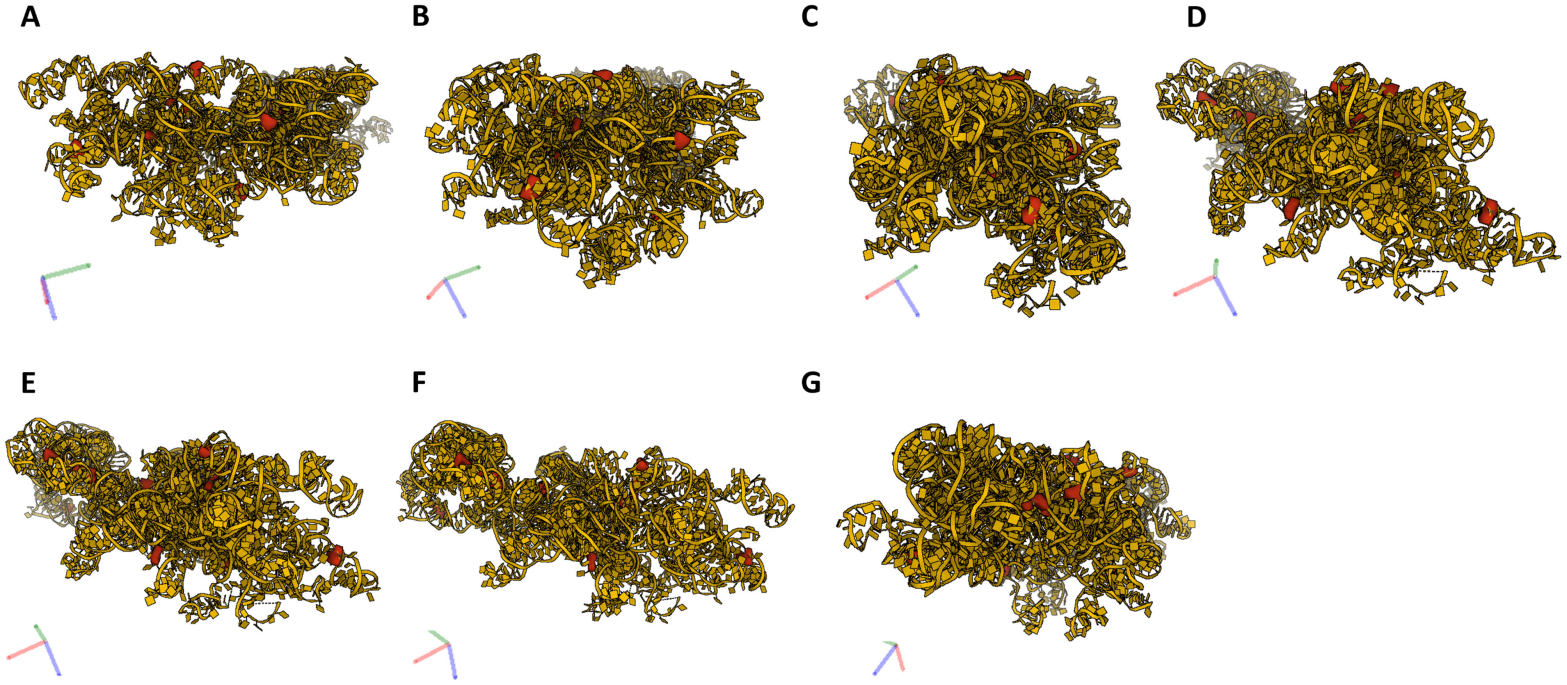
18S rRNA and its PTMs. **(A–G)** Snapshots of 18S rRNA and its PTMs (A99, U116, U406, G436, C462, A668, U686, U822, A1031, C1272, U1326, G1328, U1442, C1703) taken at different rotations. 18S rRNA (yellow), its modifications (red).

**Figure 5:**
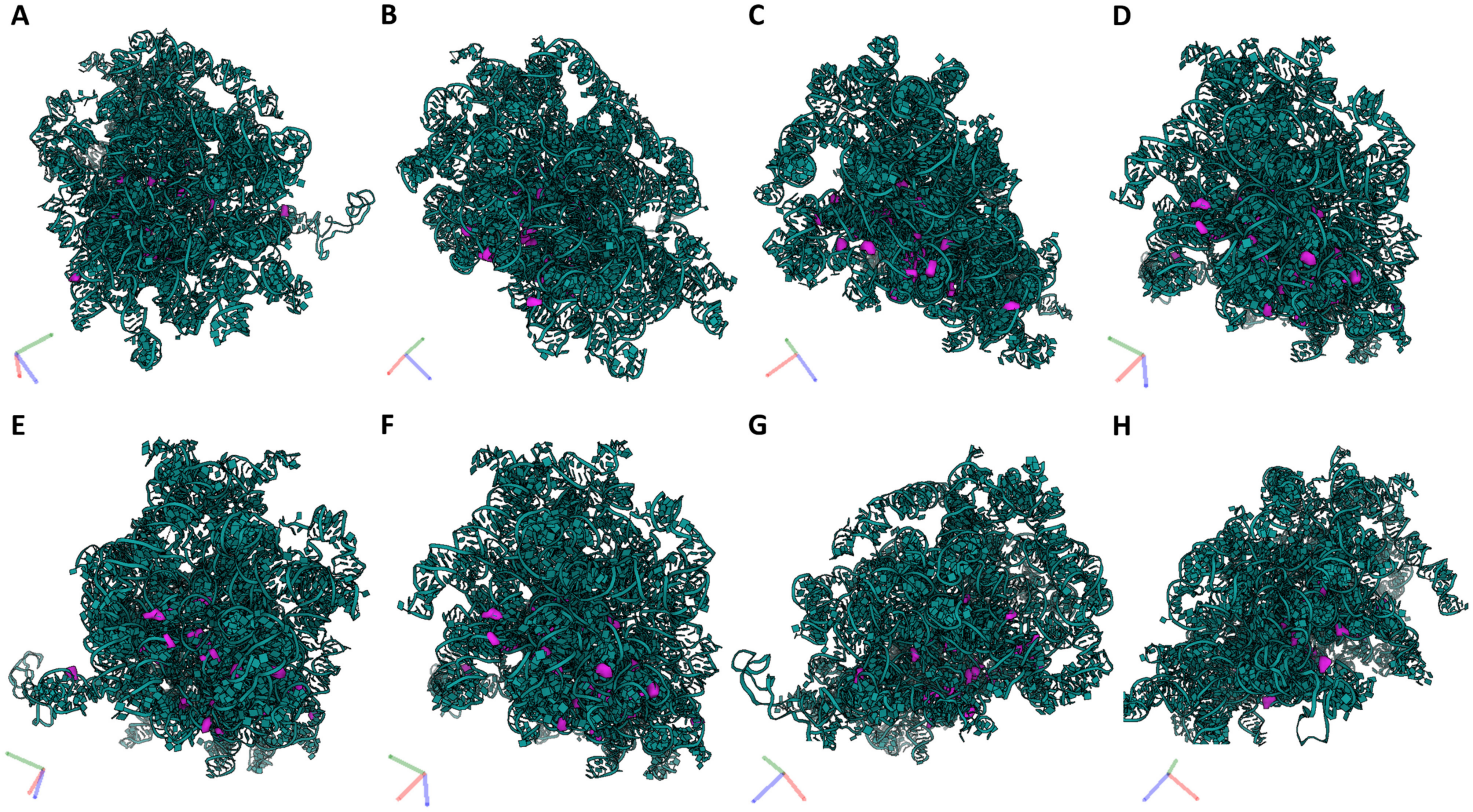
28S rRNA and its PTMs. **(A–H)** Snapshots of 28S rRNA and its PTMs (A1326, A1524, A1871, A3785, C1881, C2861, C3808, C3869, C4054, C4456, G2876, G4228, G4494, G4499, G4618, U1582, U1683, U1782, U1792, U2837, U3818, U4423, U4431, U4442, U4471, U4500, U4673) taken at different rotations. 28S rRNA (teal), its modifications (purple).

**Figure 6:**
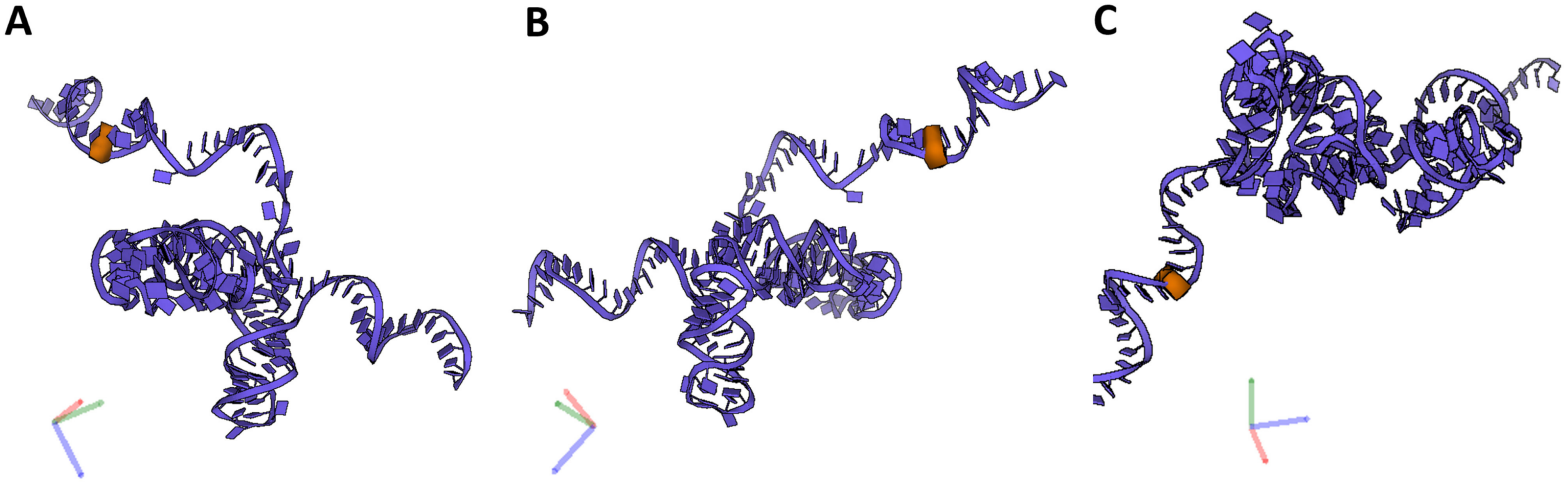
5.8S rRNA and its PTM. **(A–C)** Snapshots of 5.88S rRNA and its PTM (U14) taken at different rotations. 5.8S rRNA (blue), its modification (orange).

**Figure 7:**
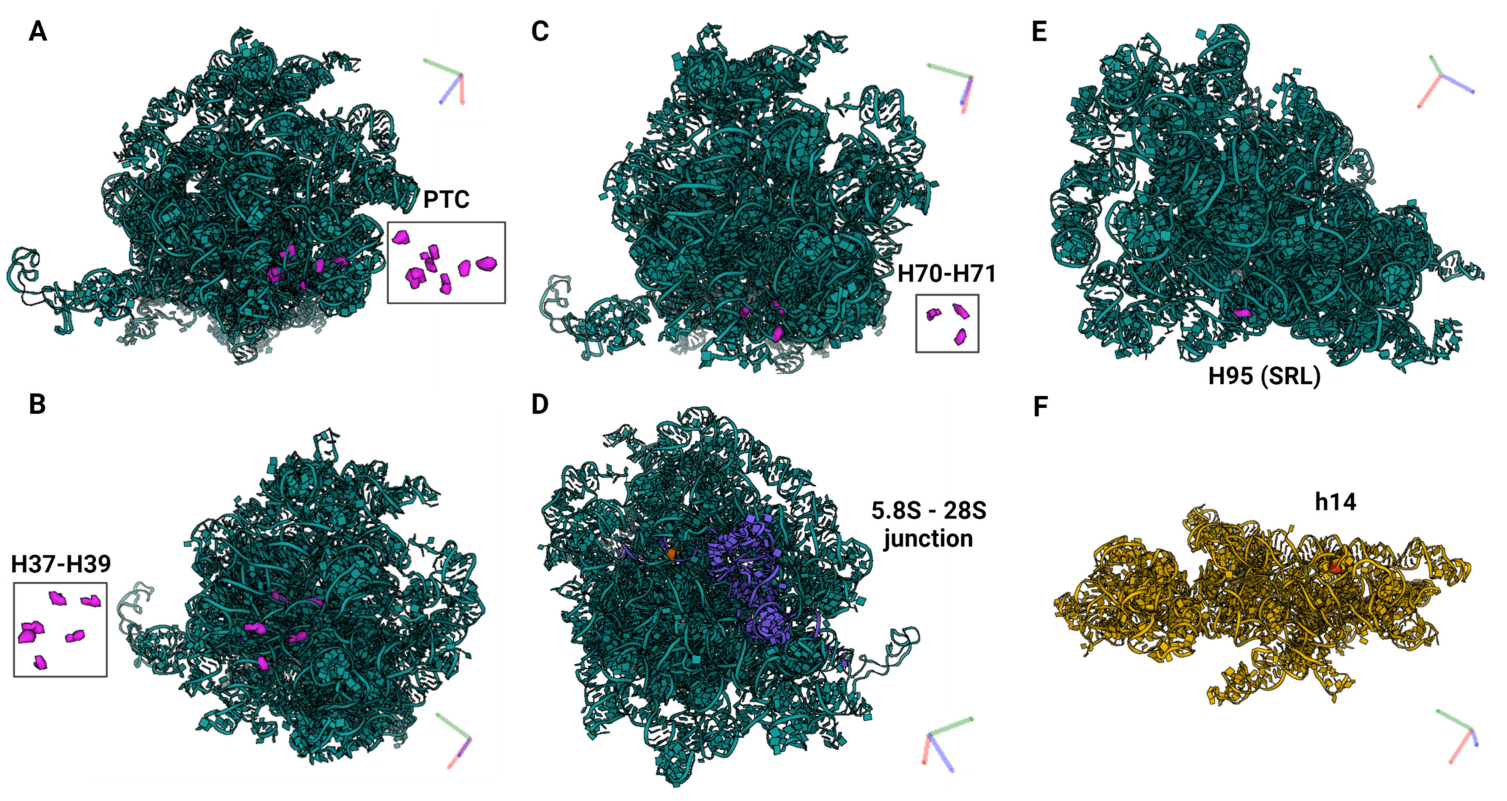
PTMs localized within functionally important regions of the ribosomes. **(A)** Eight PTMs (U4423, U4442, U4431, C4456, U4471, G4494, G4499, U4500) mapped within helices H89-91 of the PTC which catalyzes the synthesis of the polypeptide chains (34). **(B)** Six PTMs (U1683, U1782, U1792, A1871, C1881, G4228) mapped within helices H37–H39. H38 forms an “A-site finger” and the B1a SSU-LSU bridge. **(C)** Three PTMs (A3785, C3808, U3818) mapped within helices H70–H71 which are important for LSU-SSU interactions (36, 37). **(D)** One PTM (U14) located within the LSU 28S−5.8S junction and in the proximity of the polypeptide exit tunnel (PET) wall (38). **(E)** PTM G4618 mapped within H95 (also known as SRL; sarcin–ricin loop) on LSU, and **(F)** C462 within h14 on SSU. H95 and h14 interact with each other to form a part of a GTPase-associated center (GAC) which is important for peptide release (39, 40).

Of the predicted 42 PTMs, 14 mapped on 18S rRNA of the SSU (Figure 4), 27 mapped on 28S (Figure 5), and one on 5.8S of LSU (Figure 6). During the translation, the SSU monitors codon-anticodon base-pairing between the mRNA and tRNAs thus mediating the correct decoding, while the LSU, which harbors the catalytically active peptidyl-transferase center (PTC), is responsible for the synthesis of the nascent polypeptide chain (34). The majority of the predicted LSU—mapped PTMs appeared to be located within the inner regions of the ribosome (Figures 5, 6), where the PTC is located (34). In fact, a cluster of eight predicted PTMs (SNORA1-U4471; SNORA16-U4442; SNORA21-U4431, -U4500; SNORA68-U4423; SNORD49-C4456; SNORD69-G4494; SNORD75-G4499) mapped within helices H89-91 of the PTC (Figure 7A). Furthermore, a set of six predicted PTMs (SNORA7-U1792; SNORA9-U1683 and -U1782; SNORD38-A1871; SNORD48-C1881; SNORD58-G4228) located within helices H37–H39 (Figure 7B). H38 forms an “A-site finger” which is located just above the A-site. It interacts with 5S rRNA and SSU protein S13 thus forming a B1a SSU-LSU bridge (35). Additionally, three of our DE snoRNAs (SNORD10-C3808; SNORD15-A3785; and SNORD17-U3818) are predicted to target a conserved cluster of PTMs within LSU helices H70 and H71 (Figure 7C), both of which are important for LSU-SSU interactions (36, 37). Additionally, SNORD71-U14 on 5.8S rRNA located within the LSU 28S-5.8S junction (Figure 7D), in the proximity of the polypeptide exit tunnel (PET) wall (38). Two other predicted PTMs were interesting based on their localization. SNORD91-G4618 on LSU H95 (also known as SRL; sarcin–ricin loop; Figure 7E) and SNORD14-C462 on SSU h14 (Figure 7F). H95 interacts with h14 to form a part of a GTPase-associated center (GAC), which is important for peptide release (39, 40). The rest of the predicted PTMs were relatively spread along the 28S and 18S rRNAs and no other apparent clustering was observed.

## 4 Discussion

SnoRNAs are emerging as important regulators in OA (patho)biology. While several studies reported DE of snoRNAs in OA (22–24, 29), mechanisms of action by which they contribute to OA pathobiology are incompletely understood. Two recent papers provided evidence that snoRNA-mediated ribosome heterogeneity drives OA development and progression in human (25, 26). There is only very limited data on expression profiles and roles of snoRNAs in equine OA (29), particularly in relation to their functions in ribosome heterogeneity. In the present study, we used an established equine osteochondral fragment model of OA (2), and measured snoRNA levels at baseline (Day 0) and in OA (Day 28 and 70 post-OA inducing surgery) equine SF.

Several studies showed that snoRNAs are present and can be reliably detected in biofluids, including blood, plasma, serum and SF (29, 41, 42), where they are present in either free form, or encapsulated in extracellular vesicles (EVs) (43). EVs are secreted membranous vesicles facilitating intercellular communication and (patho)physiological processes throughout the body. EV's cargo comprises proteins, lipids, DNA, and various RNA species including snoRNAs, and reflects the current state of the parental cell (44–46). While plasma and serum circulating snoRNAs might reflect the systemic component of OA and thus be relevant as OA diagnostic or prognostic biomarkers (24, 30), snoRNAs present in the SF could directly contribute to OA pathobiology. The origin of SF-derived snoRNAs is still elusive but might be attributed to either controlled secretion or a passive release by damaged cells of articular joint tissues. This is the first study mapping temporal abundance patterns of snoRNAs in SF during OA initiation (Day 28) and progression (Day 70). Interestingly, while the majority of DE snoRNAs were upregulated at Day 28, at Day 70, snoRNAs were mostly downregulated. This suggests that snoRNAs might play different roles in early and late stages of OA. These results are in line with recent data on ribosome and translation regulation in OA. Studies in rodents showed that mTORC1, a critical signaling pathway regulating cellular protein synthesis, is activated most prominently in the early stages of OA (47, 48), leading to increased rates of total protein synthesis. Importantly, the changes in mTOR activity and changes in translation rates preceded morphological changes in cartilage structure during OA development (47). These mTOR/translation data are in line with a generally stablished role of mTOR in cartilage growth, development and OA (49, 50).

Here, we identified 44 snoRNAs DE in equine OA SF. Several of the snoRNAs identified as DE in equine OA SF were dysregulated in our previous OA studies, suggesting similarities in snoRNA regulation across species. For example, levels of a non-canonical snoRNA U3, decreased at Day 70 in out dataset, were also decreased in human OA chondrocytes, and this had negative effects on ribosome biogenesis and translation capacity (23). Levels of canonical SNORA71 declined in aging equine chondrocytes (51) and were also decreased in our OA SF (Day 70). Non-canonical SNORA43 and canonical SNORD38 (upregulated) were regulated in joints of OA mice following destabilization of the medial meniscus (24). Serum levels of SNORD38 were significantly elevated in patients developing cartilage damage 1 year following anterior cruciate ligament injury (30). In line with these results, we found SNORD38 to be increased in OA SF. Taken together, our results of equine SF snoRNA profiling in OA are in line with previously published data, all supporting the conclusion that snoRNAs play important roles in OA (patho)biology across species.

Nevertheless, the mechanisms of action by which snoRNAs contribute to OA pathobiology are hardly understood. While the canonical, snoRNA-mediated regulation of ribosome function and translation was experimentally validated in OA (25, 26), non-canonical mechanisms of action are yet to be explored and understood in depth. The majority of DE snoRNAs identified in this study were canonical snoRNAs predicted to guide 2′-*O*-me and ψ modifications of rRNAs. To hypothesize the roles of snoRNA-mediated ribosome heterogeneity in equine OA, we mapped these predicted rRNA PTMs within a 3D ribosome model. More than 200 rRNA nucleotides of eukaryotic ribosomes are 2′-*O*-me or ψ. As the polypeptide-generating catalytic properties of the ribosome are undertaken by rRNAs, rather than RPs (51), rRNA PTMs are expected to have a significant impact on ribosome function (13, 14). They are highly evolutionary conserved and cluster within functionally important regions of ribosomes, including the decoding and tRNA binding sites (the A-, P- and E-sites), the PTC, and the intersubunit bridges (34, 52). As a result, rRNA PTMs affect the accuracy and the efficiency of translation at the global scale, but also at the level of individual mRNAs (26, 53–57). Our rRNA PTMs mapping revealed that several important regions of the ribosome could be targeted by snoRNAs DE in equine OA.

Eight predicted PTMs mapped within LSU helices H89–H91 which form the catalytic center of the ribosome, PTC. Several previous studies demonstrated that rRNA PTMs located within the PTC are important for ribosome function as well as cellular fitness and survival (58, 59). Mistargeted 2′-*O*-me of PTC rRNA nucleotides, which are not naturally modified using engineered snoRNAs caused severe growth defects, impaired ribosome biogenesis, and a marked decrease in translation rate (58). Furthermore, yeasts depleted of 1–5 snoRNAs guiding conserved ψ within PTC were investigated. While the translation was substantially impaired in strains that lost ψ in the A site of the tRNA binding site, depletion of other ψ had subtle or no apparent effects. However, synergistic effects were observed when multiple snoRNAs were depleted (55).

Furthermore, six predicted PTMs were located within helices H37–H39. H38 forms an “A-site finger” which is located just above the A-site of tRNA binding. It interacts with 5S rRNA and SSU protein S13 thus forming a B1a SSU-LSU bridge. It also directly interacts with A-site tRNA (35). ASF is important for efficient translational activity and translation fidelity, specifically for maintaining the reading frame (35). H38 and neighboring H37 and H39 contain an unusually dense cluster of Ψ modifications. Depletion of these Ψs in yeasts negatively affected ribosome biogenesis, disrupted polysome formation, and global translation activity, and caused an overall decrease in cellular fitness and increased sensitivity to ribosome-targeting drugs (60). Three predicted PTMs mapped within helices H70–H71. Mutations in H70 of *E. coli* 23S rRNA impaired LSU-SSU interactions (37). Its localization also suggests that it may influence interactions of tRNAs at the A- and P-sites and activity of the PTC. H71 together with h44 of the SSU form an intersubunit bridge B3, important for translation (36). Similarly to H37–H39, the deletion of PTMs within H70–71 caused ribosome instability and impaired ribosome fidelity. We also mapped PTMs within h14 and H95 (also known as SRL; sarcin–ricin loop). H95 and h14 interact with each other to form a part of a GTPase-associated center. Polypeptide chain release factors eRF3 and eRF1 bind to GAC by interacting with h14 and thus form a pretermination complex, necessary for peptide release (39, 40). The deletion of H95/SRL caused defects in eRF-dependent steps of translation and a loss of EF-Tu-independent A-site tRNA binding (61). Altogether, these data indicate that differences in PTM levels of rRNA nucleotides within these important helices could potentially significantly affect ribosome functions, including translation elongation, termination, peptide release, but also influence overall translation fidelity and efficiency. Nevertheless, an experimental validation of this hypothesis is vital to draw any conclusion.

Another snoRNA that was regulated in equine OA SF was SNORD71. This snoRNA guides 2′-*O*-me of U14 on 5.8S rRNA which is located within the 28S-5.8S junction, in the proximity of the polypeptide exit tunnel (PET) wall (40). SNORD71-guided 2′-*O*-me of nucleotide U14 stabilizes the secondary and tertiary structure of 5.8S rRNA, thus affecting its conformation state and its interaction with 28S rRNA (62). Importantly, a decrease in modification levels of 5.8S-U14 was already implicated in human OA (26). In this study, healthy primary human articular chondrocytes were exposed to OA SF from patients with end-stage OA. This resulted in site-specific changes in chondrocyte rRNA PTM profiles, including a decrease in 2′-*O*-me of 5.8S-U14. Additionally, *SNORD71* KO cell pools in which ribosomes were lacking 2'-*O*-me of 5.8SU14 we generated. This affected ribosome functions such as translation modus, fidelity, internal ribosome entry site (IRES)-mediated translation initiation, and sensitivity against ribosome-targeting antibiotics. Most importantly, the loss of 2′-*O*-me of 5.8S-U14 led to an increase in the translation of collagen type I mRNA, which is a fibrotic protein associated with OA (26). In line with these findings, we found decreased levels of SNORD71 in equine OA SF. These data also support our earlier hypothesis that snoRNAs could play a role in OA by guiding rRNA PTMs within important ribosomal regions, thus regulating ribosome functions and translation in OA.

Within a joint cavity, several tissues, including articular cartilage or synovium, are in direct contact with SF and release nutrients, growth factors, signaling molecules, and EVs into the SF thus contributing to its composition (63). The DE snoRNAs identified in OA SF might therefore reflect the changes in snoRNA expression levels in joint tissues, or alternatively, come from the circulation. In line with this, a recent study reported that snoRNA expression patterns in SF-derived EVs change during progression of OA, as shown in an experimental equine OA model (43). Several of the DE snoRNAs identified in this study overlapped with our results, including SNORD15, SNORD58, U3, and others. OA-associated changes in SF composition have detrimental consequences for surrounding tissues (64–66). Importantly, exposing healthy chondrocytes to OA SF led to OA-related changes in chondrocyte phenotype and importantly, also site-specific changes in their rRNA PTM profiles (25, 26). Therefore, it is possible that DE of snoRNA within SF could affect rRNA PTMs of cells within the joint cavity. Nevertheless, the expression level of specific snoRNA and the modification level of its predicted target do not always correlate with each other (67, 68). For example, even though the knockdown of methyltransferase *FBL* in HeLa cells caused a general decrease in rRNA 2′-*O*-me levels, some sites were affected more than others. However, these changes did not directly correlate with expression levels of the corresponding snoRNAs (67). Considering this, the links between DE snoRNAs and their PTMs we proposed in this study need to be experimentally validated before we draw any definitive conclusions.

The data presented in this study indicate that snoRNAs are regulated in equine OA and we speculate that this will have consequences for ribosome function. This is based on our previous data showing that manipulation of snoRNA expression in articular chondrocytes affects ribosome function and cellular proteome (25, 26). Nevertheless, while our study provides novel insights into the differential expression of snoRNAs in equine OA and their predicted effects on rRNA-PTM-based ribosome heterogeneity, we acknowledge the lack of direct functional validation of these results to confirm their impact on ribosome activity and translation dynamics. In the future, techniques such as RiboMethSeq (69) and HydraPsiSeq (70) should be utilized to investigate changes in rRNA 2′-*O*-me and ψ profiles in equine OA in more detail. Furthermore, functional evaluation of heterogenous ribosomes using ribosome profiling or translational reporter assays is needed to determine how snoRNA-mediated rRNA heterogeneity influences ribosome function and cellular proteome in the context of OA. The development and progression of OA are fuelled by changes in protein expression programs (10, 11) and as such, translation regulation plays an important role in OA (9). Thus, an understanding of snoRNA-mediated translation regulation in OA might be valuable in developing new OA treatments in the future. Moving forward, it would be interesting to measure snoRNA levels as well as rRNA PTM profiles across different cell types of joint tissues in OA. Then we could directly link the snoRNA expression levels with the corresponding changes in their target rRNA PTMs and examine their regulation in OA in individual tissues. Experiments depleting and/or overexpressing selected snoRNAs and investigating ribosome functions would then shed light on snoRNA-mediated translation regulation and its role in OA pathobiology.

OA is a complex multifactorial and heterogeneous disease. In fact, it is becoming clear that OA represents a spectrum of conditions with distinct clinical phenotypic characteristics and underlying molecular mechanisms (endotypes) (71). Because of this, the efforts to develop a universal one-treatment-fits-all drug therapy failed in the past. A deeper understanding of underlying molecular processes and their relative contribution to particular OA phenotypes and endotypes will be important for the development of targeted OA treatments in the future. Emerging data on snoRNAs and their roles in regulating ribosome heterogeneity and translation in OA, imply that targeting snoRNA expression could be used in OA therapy. This could be achieved using intra-articular injections of antisense oligonucleotides (ASOs), or on the other hand, snoRNA-overexpressing constructs. Ribosome-targeting therapy might be novel for the OA field, but it is well recognized for treatment of other diseases (72, 73). For example, Ataluren, a small-molecule compound, is now approved by the European Medicine Agency to treat male patients with Duchenne muscular dystrophy, a disease caused by non-sense mutations in the dystrophin gene (73, 74). Ataluren interacts with ribosomes and facilitates the recruitment of near-cognate tRNAs, thus allowing for readthrough of premature stop codons in the dystrophin mRNA (75). Furthermore, many potential therapeutic agents and small molecule inhibitors targeting ribosome biogenesis, translation initiation, or specific “onco-ribosomes” are being tested in cancer clinical trials (76, 77). Importantly, as discussed earlier, OA patients might also benefit from translation- and ribosome-targeting therapy. Overall, future research focused on a comprehensive understanding of the translation dynamics in OA might aid in developing new, exciting OA treatment strategies.

## Data Availability

The datasets presented in this study are deposited in ENA SRA repository, accession numbers, PRJEB91727, ERP174663.
