# Supplementary material for: Exploring the roles of snoRNA-induced ribosome heterogeneity in equine osteoarthritis
Source: Front Vet Sci. 2025 Jul 10;12:1562508. doi: 10.3389/fvets.2025.1562508 (PMC12288042; doi:10.3389/fvets.2025.1562508)
Supplement: Supplementary file 1 [file Table_1.docx]

**Glossary**

**Antisense Oligonucleotide (ASO)**: a short, synthetic, single-stranded oligodeoxynucleotide, complementary to selected target RNA. By binding the target RNA, ASO transiently regulates its expression by blocking translation, causing degradation, or regulating splicing.

**Biomarker:** a characteristic or a molecule that can be objectively measured and evaluated as an indicator of normal or pathological biological processes, or pharmacologic responses to a therapeutic intervention. Accuracy, precision, high sensitivity, and specificity are important characteristics of an ideal biomarker.

**Extracellular vesicles (EVs):** a heterogeneous group of secreted membranous vesicles facilitating intercellular communication and (patho)physiological processes throughout the human body. Based on their size, content, and biogenesis and release pathways, EVs are subcategorised into three main subtypes: exosomes, microvesicles and apoptotic bodies. EV cargo comprises proteins, lipids, DNA, and various RNA species, including snoRNAs, metabolites and whole organelles. Importantly, the process of EV production, loading and release by the parental cell is cell-type and condition-specific.

**Ribosome Heterogeneity**: a term describing the fact that not all ribosomes have the same composition as a result of rRNA sequence variation, rRNA post-transcriptional modifications, incorporation of ribosomal protein paralogs, alterations in RP stoichiometry, and RP post-translational modifications. Ribosome heterogeneity has been documented at the level of different species, developmental stages, tissues within a single cell, in disease or distinct growth conditions.

**Ribosomal RNA post-transcriptional modifications (rRNA PTMs):** chemical changes made to rRNA molecules after they are transcribed. These modifications are essential for ribosome biogenesis, structure, and function, particularly for ensuring accurate and efficient protein synthesis. The two most common rRNA PTMs are **2’-*O*-methylation** (2’-*O*-me; when a methyl group is added to the 2′-hydroxyl of the ribose sugar), and **pseudouridylation** (ψ; when uridine is converted into pseudouridine).

**Ribosome Specialisation**: a concept in which ribosome heterogeneity confers functional ribosome specialisation, contributing to translational control. The origin of the ribosome specialisation theory dates back to the 1950s, however, the compelling evidence of functional specialisation of ribosomes emerged relatively recently.
